# Behavioral Assessment of Six Reptile Species during a Temporary Zoo Closure and Reopening

**DOI:** 10.3390/ani12081034

**Published:** 2022-04-15

**Authors:** Jennifer Hamilton, Kylen N. Gartland, Megan Jones, Grace Fuller

**Affiliations:** Center for Zoo and Aquarium Animal Welfare and Ethics, Detroit Zoological Society, Royal Oak, MI 48067, USA; kgartland@dzs.org (K.N.G.); mjones@dzs.org (M.J.); gfuller@dzs.org (G.F.)

**Keywords:** reptile welfare, visitor effects, COVID-19, zoo animal behavior

## Abstract

**Simple Summary:**

Reptile welfare in captivity is vastly understudied given the diverse taxa and the large number of individuals held in zoos and aquariums. The varied natural ecologies of reptiles have the potential to impact how they perceive different stimuli, including zoo visitors. The current study aimed to explore the impact of visitors through observations on small groups of six reptile species during a temporary zoo closure due to the COVID-19 pandemic by measuring behavioral diversity, use of enclosure space, and select behaviors. The majority of the species showed intermediate responses to the change in visitor presence that varied in valence; however, some responses were more pronounced.

**Abstract:**

Although reptiles are commonly housed in zoos and aquariums, their welfare is understudied for the diversity of species housed and the taxon’s current captive population size. The sensory abilities of reptiles have adapted to the varied ecological niches they inhabit, and these evolutionary adaptations impact how reptiles perceive the stimuli around them—including zoo visitors. This study aimed to assess visitor effects on small groups of six reptile species during a temporary zoo closure due to COVID-19 by measuring behavioral diversity, use of space (measured by a spread of participation index), and select behaviors. The species assessed showed diverse responses. The Catalina Island rattlesnakes (*Crotalus catalinensis*) demonstrated increased investigation and behavioral diversity after the zoo reopened compared to when the zoo was closed, but the European glass lizards (*Pseudopus apodus*) showed decreases in the amount of time spent exposed to the observers’ view and in their evenness of space use after the zoo was reopened to visitors. The other species, including beaded lizards (*Heloderma horridum*), Sonoran spiny-tailed iguana (*Ctenosaura macrolopha*), Arrau turtles (*Podocnemis expansa*), and dwarf caimans (*Paleosuchus palpebrosus*), had intermediate changes in their responses to visitor presence.

## 1. Introduction

The sheer number of reptile species housed in captivity is not reflected in the number of publications on their welfare. In 2011, 445 different species of reptiles were listed as being housed globally by zoos and aquariums (hereafter zoos), representing roughly 11% of the total terrestrial vertebrate species held by zoos [1]. However, only three percent of welfare publications between 2008 and 2017 focused on reptiles [2]. Evidence that reptiles experience anxiety and pleasure has garnered only one paper on reptile sentience for every 50 papers published on mammal sentience [3]. The gap in knowledge for the diverse reptile taxon has the potential to limit opportunities for great welfare in zoos.

Reptiles have evolved to live in a variety of ecosystems and temporal niches. Some inhabit open areas with few hiding spots such as the Catalina Island rattlesnakes (*Crotalus catalinensis*) [4] and Sonoran spiny-tailed iguanas (*Ctenosaura macrolopha*) [5]. Others prefer closed habitats where vegetation allows ample hiding places, such as dwarf caimans (*Paleosuchus palpebrosus*) [6]. Some individuals are labeled as nocturnal (e.g., Catalina Island rattlesnakes [7]), others diurnal (e.g., European glass lizards, *Pseudopus apodus*, [8]), and others have more flexible activity cycles. Beaded lizards (*Heloderma horridum*) are active at night during the rainy season and diurnal at other times of the year [9], and diurnal Arrau turtles (*Podocnemis expansa*) tend to complete behaviors related to nesting at night [10]. Reptile diets span the gamut from herbivorous to carnivorous and can vary depending on life stages [11,12,13]. Stratum use of reptiles also varies, with taxa described as semi-fossorial [8], aquatic [6], arboreal [9], and ground-dwelling [4].

Reptile sensory abilities are as varied as the ecological niches they inhabit, and these variations influence both their ability to process stimuli and the perceived intensity of the stimuli in a captive environment. Diverse locales enabled reptile vision to evolve to meet each species’ needs. For example, a comparison of visual systems between the freshwater crocodile (*Crocodylus johnstoni*) and the saltwater crocodile (*Crocodylus porosus*) demonstrated that the freshwater crocodile is more sensitive to the longer wavelengths of light that are more prevalent in freshwater habitats [14]. Olfaction has undergone numerous functional adaptations across divergent evolutionary lines within the broader reptile taxa. Tongue-flicking is often found in snakes and lizards and is a way for chemosensory stimuli to be brought to the vomeronasal organ [15]. Crocodilians do not have a vomeronasal organ, but data suggest they scent mark and can locate food by scent [16]. In addition, reptiles have evolved diverse ways of experiencing noise and vibrations. Many lizards have tympanic ears [17], crocodilians rely on integumentary sensor organs to process pressure and vibrations [18], and snakes utilize somatic hearing or the detection of air and ground-borne vibrations using the body’s surface [19]. Variations in reptiles’ sensory capacities also limit generalizations of responses to complex stimuli such as visitor effects across the entire taxa.

Visitors can present potentially overwhelming visual, auditory, and olfactory stimuli that may be disconcerting to zoo animals with sensory abilities adapted to wild environments. Occasionally visitors also may engage in inappropriate behaviors such as throwing items, yelling, using flash photography, and tapping on glass [20,21]. In lab settings, photo flashes have been observed to suppress normal metabolic processes, while tapping on the glass temporarily lowered the rate of oxygen consumed in Mozambique tilapia (*Oreochromis mossambicus*) [22]. High levels of anthropogenic noise have been related to increased cortisol levels and other physiological effects across a variety of taxa, including reptiles [23]. Although most research has shown that inappropriate visitor behaviors occur at relatively low rates, the potential welfare concerns caused by these disturbances should still be mitigated when possible [20,21,24]. The potential impact of zoo visitors has led to increasing research to assess their effect on animals in captivity [25]. However, visitor effect studies have mainly focused on mammals, and even more specifically primates and carnivores, making inferences to the impact on other taxa difficult, especially reptiles and amphibians.

The unexpected temporary closures across many institutions due to the COVID-19 pandemic allowed for new opportunities to pursue visitor effect research in some of these understudied taxa. Riley et al. [26] found relatively few changes in the behavior of a group of male Nile crocodiles (*Crocodylus niloticus*) between 2020 (zoo closed) and 2019 (zoo open). They noted more frequent agonistic behavior and fewer individuals in social contact during the zoo closure as opposed to normal operations, but these changes were small and also impacted by time, date, and temperature-related variables. Research on 13 reptile species found species-specific differences in activity and visibility related to the presence and absence of visitors [27]. Tokay geckos (*Gekko gecko*), in particular, were less visible when visitors were present compared to when the facility was closed to visitors. Similar trends in visibility during a temporary COVID-19 pandemic closure and reopening were observed for amphibian species [28]. The majority of amphibian species observed decreased their visibility when visitors returned compared to when the facility was closed, although there was evidence of habituation to visitors over time. One species, the smooth newt (*Lissotriton vulgaris*), displayed extremely low visibility during and after the closure and was eventually removed from the study, causing the researchers to contemplate whether there is a threshold where a species’ lack of visibility indicates a lack of habituation to captivity. Overall, the differing responses to visitors by various reptiles and amphibians may be due to factors related to their natural ecology or variables specific to their captive environments. Further research could help identify factors influencing visitor effects on reptiles and amphibians and grow our understanding of how visitors affect welfare.

This study’s original goal was to explore visitors’ impact on reptiles, as at the time of design, limited research was available. We hypothesized that visitor presence would have no impact on the behavior, evenness of space use, or behavioral diversity of the species chosen. Six species of reptiles with diverse natural ecologies were selected with the expectation that natural history could impact their behavior, including a single Sonoran spiny-tailed iguana and small groups of Catalina Island rattlesnakes, European glass lizards, beaded lizards, Arrau turtles, and dwarf caimans.

## 2. Materials and Methods

This study was reviewed and approved by the Animal Welfare and Management Committee of the Detroit Zoological Society (DZS).

### 2.1. Subjects and Housing

This study occurred in 2020 when the Detroit Zoo was closed to visitors due to the COVID-19 pandemic (April to mid-June) and after reopening with limited capacity (mid-June to July). Observations were conducted on six species in four enclosures. We selected enclosures based on the species’ natural ecologies (Table 1), conversations with care staff, and a review of annual animal welfare assessments (i.e., a survey of a group or individual’s inputs and outputs completed annually; see [29] for an example). We also considered their ability to avoid visitors and their tendency to direct attention towards visitors. There were no changes to animal care practices between the study conditions. However, care staff was present in the building an additional hour when the zoo reopened compared to when the zoo was closed (Zoo Closed: 08:00 to 17:30; Zoo Open: 08:00 to 18:30). All animals were fed 2–3 times a week except for the rattlesnakes, which were fed once every two weeks. The iguana and turtles were offered a mix of vegetables (e.g., greens, carrots, sweet potato). All individuals were offered appropriate protein sources (e.g., insects, fish, rodents) according to their natural ecology. Lights in the visitor area of the building were on from 08:00 to 17:30 while the zoo was closed and 08:00 to 18:30 when the zoo reopened. We did not monitor the temperature of the building during these observations; however, the building temperature was monitored from September to November 2020 and April to June 2021. The temperature range recorded during both of these periods was from 23.3 °C to 27.7 °C with an average ± standard error (SE) of 25.4 ± 0.1 °C.

Four of the observed species lived in covered enclosures with gunite walls and glass fronts (Enclosures A, B, and C; See Table 2). Enclosures A and C consisted of rock and sandy substrates, and both had artificial cacti, sticks, and caves as furniture. Enclosure B had soil and mulch as substrates with evergreen branches as furniture. Habitats A, B, and C were approachable by visitors during the Zoo Open condition. The last enclosure (Enclosure D), which housed the turtles and caimans, was an open-top enclosure with a glass front and gunite walls. Enclosure D had a 6400-L pool, a sandy substrate on land, and a rock waterfall and ledges accessible to the animals. Live plants were incorporated into the enclosure. On 11 May, a tree was removed from the enclosure to give the animals more land space. Time spent engaged in individual behaviors and space use were compared before and after this enclosure modification, and no significant differences were seen. As such, observational data used for this research began on 12 May to limit the number of confounding factors. Enclosure D was also not approachable by visitors during the Zoo Open condition, as stanchions kept visitors about 0.76 m from the front of the enclosure. None of the enclosures included heat lamps or heat mats during this time frame. All enclosures had primary light fixtures set on a 12/12 light/dark cycle during the study. Additional light entered enclosures from the visitor area and skylights in the center of the building.

### 2.2. Behavioral Observations

For all animals except the glass lizards, observers simultaneously monitored all animals in one enclosure using focal scan sampling [33]. Observers identified each individual using unique physical characteristics. Focal observations lasted for 15 min and were conducted twice per day, once in the morning and once in the afternoon. Identification of the European glass lizards was unreliable, so this species was monitored using group scan sampling following the same observation schedule. We randomized enclosure observation order and balanced observations such that enclosures were observed equally across four time periods: 08:00 to 10:00, 10:00 to 12:00, 12:00 to 14:00, and 14:00 to 16:00. Observations occurred for 18 nonconsecutive days within the Zoo Closed condition and 18 nonconsecutive days after the zoo reopened with a daily limit of 2900 visitors (Table 3). One day (6% of days) was removed from the European glass lizard dataset during the Zoo Open condition due to routine veterinary physicals that removed individuals from the enclosure. Two days (11% of days) were removed from the Arrau turtles and dwarf caiman dataset during the Zoo Closed condition due to more than 50% of the pool being drained for cleaning.

Observers used the ZooMonitor app for data collection [34]. Observations were conducted live. Although the observers aimed to stay five feet from the front of the habitat during each observation, they were a constant presence during both conditions. Observers scored each animal’s behavior (Table 4), exposure, and location in the enclosure at one-minute intervals. Additionally, all occurrences of select behaviors and visitor-glass interactions were recorded. Authors JH and MJ collected all the data and demonstrated >90% inter-observer reliability based on the percent difference in behaviors scored across three observations. Observations considered for inter-observer reliability had to include three or more different behaviors, and individuals had to be visible for more than 50% of the observations. Cohen’s Kappa for behavior, as calculated within the ZooMonitor App, was equal to or above the recommended 0.81 for all inter-observer reliability sessions [35].

### 2.3. Data Analysis

We were interested in exploring the impact of the zoo closure on behavior (specifically social behaviors, investigative behaviors, inactive behaviors, and time exposed when visible), visibility, behavioral diversity, and evenness of space use. Additionally, we wanted to assess the relationship between visitor-glass interactions and reptile visibility. We were also interested in the relationship between exposure and visitor-glass interactions. We calculated behavioral diversity on a weekly scale using the Shannon–Wiener Index (H) [38]. This diversity index was designed to measure both richness and equality of distribution, in this case of behaviors, within a larger repertoire. All positive, active behaviors were included in the index, including social, move, eat, investigate, and others, as suggested by Miller et al. [39]. However, we calculated the proportion of each behavior using a count of all visible behaviors as the divisor to account for the large amount of time spent inactive. Behavioral diversity was calculated on a weekly timeframe to account for the days when the reptiles only displayed one behavior. The index is calculated as

$$H_{i\;=\;1}^{S}=-\sum^{\;}p_{i}\ln\left(p_{i}\right)$$

where in *p_i_* represents the frequency of behavior *i* in the repertoire, *S* is the number of behaviors within the repertoire, and *H* is the maximum value for a given community calculated as *H_max_* = ln*S*. Behavioral diversity is measured on a continuum starting at 0, representing low behavioral diversity. Higher values represent greater behavioral diversity. The maximum *H* can only be reached if all active behaviors are equally represented [38]. The Shannon–Wiener Index has been used across a multitude of zoological studies and adapted for various hypothetical tests [37,39,40,41,42,43].

We calculated the evenness of space use on a weekly scale using Dickens’ Spread of Participation Index (SPI) [44]. The weekly scale was used to account for the multiple days when the reptiles used only one location. This index has been widely used in zoos to measure the evenness of enclosure use by individuals or social groups [45,46,47,48]. The Dickens’ SPI divides a given enclosure space into equal zones and calculates a value that varies between 0 and 1. In the case of SPI, 0 represents maximum enclosure use, while 1 represents minimum or restricted enclosure use. As such, increased SPI represents lower enclosure use. The SPI is calculated as

$$\mathrm{SPI}\;=\frac{M\left(n_{b}-n_{a}\right)+\left(F_{a}-F_{b}\right)}{2\left(N-M\right)}$$

where in *N* represents the total number of observations across all zones, *M* is the mean frequency of observations per zone, *n_b_* is the number of zones with observations < *M*, *n_a_* is the number of zones with observations > *M*, *F_a_* is the total observations in all zones with observations > *M*, and finally *F_b_* is the total observations in all zones with observations < *M* [44,47]. We subdivided each two-dimensional (2-D) enclosure map into 32 equal-sized 2-D zones.

To analyze behaviors of interest (social, investigate, inactive, time exposed when visible), we calculated daily percentages corrected for total visibility. To assess visibility, we calculated daily percentage visible out of total time observed. Visitor-glass interactions were calculated as a rate per day. All further analyses were conducted using SAS©, 9.4.1 (Cary, NC, USA). We used the UNIVARIATE procedure to conduct Kolmogorov–Smirnov tests for normality for each of our outcome variables. The data were non-normally distributed with high skew; therefore, we elected to perform non-parametric analyses. We used inferential statistics adapted for small samples sizes to gain a better understanding of how these groups were affected by the zoo closure; however, it was not our intention to extrapolate these results to a broader study population.

We sorted the data according to species. We then tested for variation in our outcome variables between zoo status conditions using the NPAR1WAY procedure to run Wilcoxon two-sample tests. Following previously established methodology, we corrected for small sample size by generating the test statistic using a Monte Carlo sampling method (10,000 permutations) [49,50,51,52]. Only results that had an adjusted Monte Carlo significance (Pr > |S-Mean|) of *p* < 0.05 were considered significant. We used Spearman rank correlations to explore potential relationships between visitor-glass interaction and visibility, as well as time exposed when visible. For Enclosure D, a visitor-glass interaction was only recorded once during the entire Zoo Open period, so we did not look at these relationships for the Arrau turtle and dwarf caiman. Exposure and visibility data for the Sonoran spiny-tailed iguana did not contain sufficient variation for reliable correlation testing. Similarly, limited variability in exposure for the beaded lizards negated use of a correlation. Data were sufficient for correlation analyses for all other study species.

## 3. Results

Due to the large number of descriptive and inferential statistics, all statistics can be found in Table 5 (behavior variables), Table 6 (visitor-glass interaction), Table 7 (behavioral diversity), and Table 8 (evenness of space use).

### 3.1. Catalina Island Rattlesnake

**Figure 1:**
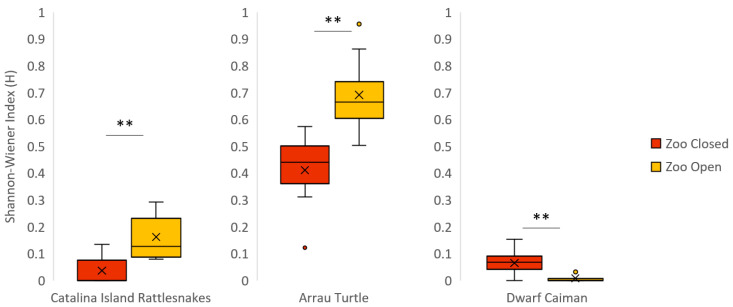
Boxplot for all significant differences within a species in the Shannon–Wiener Index (H) when the zoo was closed vs. open. For H, lower values indicate a lower variety of behaviors. The mean of each condition is represented by an “×”. Significant differences of Pr > |S-Mean| equal to 0.01 or below are indicated with a double asterisk (**).

The Catalina Island rattlesnakes displayed significantly decreased social behaviors when the zoo was closed compared to when the zoo was open (Table 5). In both conditions, the male rattlesnake was the main initiator of social interactions, which consisted of the male stroking the female with his head and tail locking. We observed the same pattern for investigative behaviors and behavioral diversity (Table 7 and Figure 1). In addition, we observed a significant decrease in inactive behaviors when the zoo opened to the public. There was no significant variation in visibility, exposure, or evenness of space use between zoo conditions. There were also no significant correlations between visitor-glass interaction and either visibility or time spent exposed when visible.

### 3.2. European Glass Lizard

**Figure 2:**
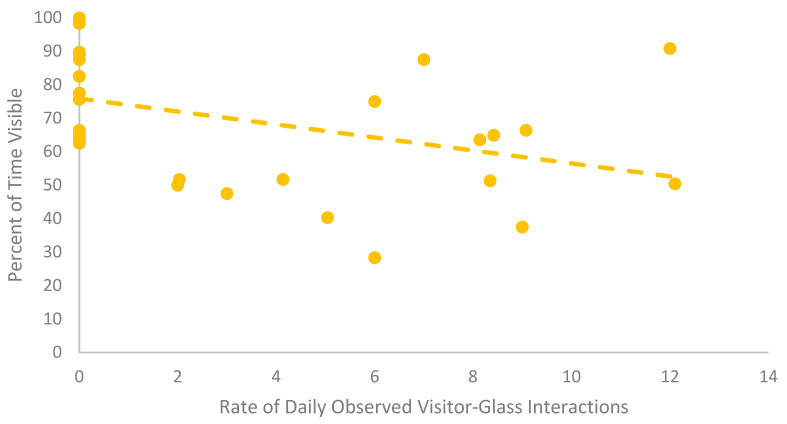
Scatterplot with a trendline comparing the daily rate of visitor-glass interaction and the daily percent of time visible for the group of European glass lizards. Each point represents a single day for the group.

**Figure 3:**
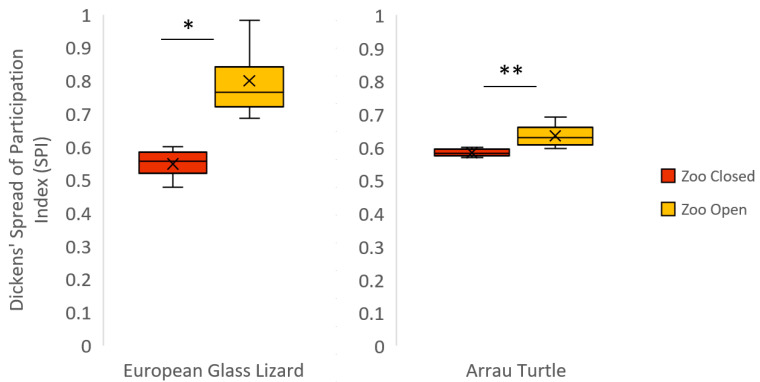
Boxplot for all significant differences within a species in the Dickens’ Spread of Participation Index (SPI) when the zoo was closed vs. open. For SPI, lower values mean more even space use. The mean of each condition is represented by an “×” and all outliers are indicated by circles. Significant differences (Pr > |S-Mean| below 0.05) are indicated with an asterisk (*), and Pr > |S-Mean| of 0.01 or below are indicated with a double asterisk (**).

The European glass lizards were never observed engaging in social behaviors. Visibility and time spent exposed when visible were both significantly different based on zoo condition. The lizards were less visible when the zoo was open (Table 5). In addition, the lizards spent more time exposed when visible in the Zoo Closed condition. There was a significant negative correlation between time spent visible and rate of visitor-glass interactions (Table 6, Figure 2). The higher the daily observational rate of visitor-glass interactions, the less time the European glass lizards spent visible. The European glass lizards also spent less time exposed when visible when the daily observational rate of visitor-glass interactions was higher (Table 6). SPI was significantly higher when the zoo was open to the public (Table 8 and Figure 3), signifying a decrease in space use after the zoo opened. The European glass lizards decreased their use of the space around the front of the enclosure in the Zoo Open condition. There was no significant variation in investigative or inactive behaviors or behavioral diversity between zoo conditions.

### 3.3. Beaded Lizard

The beaded lizards spent less time engaged in social behaviors when the zoo was closed as opposed to when the zoo was open (Table 5). The majority of the social behavior consisted of breeding-related behaviors, such as mounting. The beaded lizards displayed no significant variations in investigative behaviors, inactivity, visibility, time spent exposed when visible, behavioral diversity, or evenness of space use between zoo conditions. There was also no significant correlation between visitor-glass interactions and visibility.

### 3.4. Sonoran Spiny-Tailed Iguana

The Sonoran spiny-tailed iguana’s social behavior was infrequent and never recorded during an interval scan. Time spent exposed when visible was significantly higher when the zoo was closed to the public (Table 5). There was no significant variation in investigative behaviors, inactive behaviors, visibility, space use, or behavioral diversity between zoo conditions.

### 3.5. Arrau Turtle

Inactivity for the Arrau turtles was significantly lower when the zoo was closed than when the zoo was open (Table 5). When the zoo was closed, the turtles were in almost constant motion, swimming throughout their enclosure. When the zoo was open, the turtles displayed greater behavioral diversity (Table 7 and Figure 1). However, the increase in SPI between zoo conditions indicates a significant decrease in overall space use when the zoo was open to the public (Table 8 and Figure 3). There was no significant variation in social behavior, investigative behavior, visibility, or time spent exposed when visible between zoo conditions.

### 3.6. Dwarf Caiman

Inactive behaviors were significantly higher when the zoo reopened (Table 5). Behavioral diversity was significantly lower when the zoo reopened (Table 7 and Figure 1). There was no significant variation in social behaviors, investigative behaviors, visibility, time spent exposed when visible, or space use between zoo conditions.

## 4. Discussion

The responses of the reptiles in this study to the zoo reopening ranged in both strength and valence, although no overtly negative changes in behavior such as increased interactions with the glass or startling and freezing behaviors were observed. The European glass lizards and the Catalina Island rattlesnakes demonstrated the most pronounced behavioral responses. The European glass lizards showed a decrease in multiple behavior variables, including evenness of space use, time spent exposed when visible, and time spent visible. The decreases in both time spent exposed when visible and evenness of space use observed for the European glass lizards were consistent across all weeks of the Zoo Open condition. In contrast, the Catalina Island rattlesnakes showed an increase in investigative behavior and behavioral diversity, both behaviors with the potential to be positive indicators of welfare with further validation. Other reptiles showed limited responses to visitor presence with mixed or minor changes in behaviors observed similar to other reptile visitor effect studies [26,27]. The primary behavioral change in the beaded lizards consisted of increased social interactions likely related to breeding. In the same enclosure, the Sonoran spiny-tailed iguana’s only behavior change was decreased time exposed when visible in the Zoo Open condition. The dwarf caimans and Arrau turtles increased the amount of time they spent inactive after the zoo reopened, but whereas the dwarf caiman’s behavioral diversity decreased, the Arrau turtles’ behavioral diversity increased.

This increase in behavioral diversity for the Arrau turtles, particularly in conjunction with an observed increase in inactivity, raises questions as to the value of behavioral diversity as a behavioral indicator of welfare in this context. The turtles spent the majority of their time swimming during the Zoo Closed condition, resulting in a low behavioral diversity index score. During the Zoo Open condition, the Arrau turtles spent more time resting by the front of the enclosure and demonstrated a non-significant increase in social and investigating behaviors. The lower proportion of time spent moving, as well as non-significant increases in other behaviors, resulted in a significant increase in the Arrau turtle’s behavioral diversity. As suggested by Miller et al. [39], we did not add the proportion of inactive behavior into the behavioral diversity equation. However, we felt it was necessary to account for inactivity in the larger behavioral repertoire we used to calculate the proportions due to the great amount of time reptiles often spend on this behavior. As the behavioral diversity calculations are not weighted in favor of which behaviors are important to an individual [53], a substantial amount of time inactive could inadvertently result in what is essentially an artificially inflated measure of behavioral diversity. A measure that does not account for natural ecology can result in statistics that may not be biologically relevant to the species in question. For instance, the dwarf caimans’ significant decrease in behavioral diversity resulted from a less than two percent decrease in active behaviors. The varying indices developed for assessing behavioral diversity are likely biased by the predominant focus on more active species such as chimpanzees (*Pan troglodytes*) [54]. Behavioral diversity has the potential to be a helpful measure of welfare as it presents a composite score for the behaviors shown by an individual, but more research is needed to determine if it is a meaningful indicator of welfare when inactivity encompasses a large portion of an animal’s activity budget. This concern may also be applicable to other composite behavioral indices such as SPI.

There are a variety of indices developed for the assessment of space use, many of which differ in their method of spatial division. Plowman [47] found that dividing an enclosure based on resources avoids overestimation of space use with fewer zones required. This methodology may be more applicable for species in which ample data is available for assessing the value and preference of various resources. Such data are not widely available for reptile species. Other methods rely on an even distribution of space, which may be more appropriate for spaces with more uniform resource allocation or understudied taxa [55]. However, one might not expect equal usage of space if preferred temperatures are not equally distributed across the enclosure, calling into question the utility of SPI as a welfare indicator for heterothermic species. We are unable to address this concern as we did not measure thermal gradients in this study. We also acknowledge that some of Plowman’s concerns with the original Dickmans’ SPI, such as the accuracy of measuring based on a grid pattern and the meaningfulness of a grid in respect to the animals, could still be valid, especially when using 2-D maps wherein equal 2-D zones may not equate to equal 3-D zones. As we were only able to account for two dimensions in space, we structured the photographic enclosure maps to include all options available to the reptiles, which potentially impacted our results. As this was an exploratory study, encapsulating as many options on the map as possible was important for assessing the meaningfulness of different factors to the reptiles. The European glass lizards are a good example, as they decreased the evenness of their space use by decreasing their time spent near the front of the enclosure, potentially suggesting that the space near the front may have been less comfortable when the zoo was open. However, this was not a consistent reaction across all the animals in this study. The Arrau turtles showed a decrease in the evenness of their space use, which corresponded to the increase in the time they spent inactive in one location at the front of their enclosure, but Enclosure D had a barrier preventing visitors from approaching the enclosure’s glass front. The Catalina Island rattlesnakes, beaded lizards, Sonoran spiny-tailed iguana, and dwarf caimans all showed no change in the evenness of their space use.

Another essential element of space use to consider, particularly when investigating questions surrounding visitor effects, is the use of covered or obscured spaces. The opportunity to remove themselves from situations that cause stress by utilizing a hide or visual barrier is critical to reptile welfare and is, therefore, an essential feature of reptile enclosures [56]. When given an enriched enclosure with multiple hide options, corn snakes (*Pantherophis guttatus*) spent more time out in the open and in more relaxed body posture when compared to their standard enclosure, suggesting increased comfort in the enriched enclosures [57]. In addition, various amphibians and reptiles showed decreased visibility when facilities were open compared to during COVID-19 closures [27,28]. We recorded exposure (i.e., how much of the focal’s body was uncovered) separate from visibility (i.e., was the focal visible) because this factor may be related to different comfort levels. The European glass lizards’ decreased visibility and time spent exposed when the zoo reopened could be related to comfort in their enclosure. However, species-specific differences also need to be taken into account. Animals from more closed habitats, such as the European glass lizards, may move to a more hidden location as a strategy when processing or adjusting to new stimuli. As Boultwood et al. [28] discussed, even with species variation in visibility, consistently low visibility may indicate an inability to habituate to the zoo environment. Of our study species, European glass lizards spent the most time not visible when the Zoo was open (42.09% of the time). The rest of the species observed spent less than 2% of the time not visible, suggesting relative comfort of these individuals in the zoo setting.

Boultwood et al. [28] suggest that predator defense and enclosure fit may also factor into amphibian visibility during periods of visitor presence. Both the Catalina Island rattlesnakes and beaded lizards use venom when capturing their prey. Although not strictly a predator defense, being venomous may impact how these species react around perceived predatory stimuli, such as zoo visitors. Both species displayed primarily neutral responses to the zoo reopening. Research on beaded lizards during a similar closure at the London Zoo showed an increase in time spent visible when visitors were present [27]. The behavior between both groups of beaded lizards points to primarily neutral responses, and any differences in response may be related to individual or enclosure factors. Different enclosure features could provide species with the ability to cope with perceived visitor stimuli through visual or auditory barriers and the ability to choose proximity to visitors. This study was not designed to assess the fit of the zoo enclosures for the reptiles they housed as each species was only evaluated in one enclosure, and further research is needed to address that factor in relation to visitor impacts on reptiles.

This study focused on the larger picture of visitor presence on the welfare of reptiles, but visitor density and behavior may be important factors to consider. Jones et al. [58] found red kangaroos (*Macropus rufus*) increased their time spent in proximity to conspecifics and decreased the evenness of their space use with increasing crowd sizes. Furthermore, a selection of primates showed an increase in aggression with an increasing number of visitors [59,60]. Visitor behavior may also be an impactful factor depending on how a species perceives sensory stimuli. Crouching in front of small primate enclosures was related to a decrease in aggression compared to visitors standing at their full height [59]. The current study highlights a negative visitor behavior in interactions with the glass. The European glass lizards showed decreasing visibility and time spent exposed with increasing rates of visitors interacting with the glass. This study cannot separate whether it was a specific stimulus from this interaction (e.g., visual, auditory, tactile, proximity) or the accumulation of all of these factors resulted in the observed behavioral shifts. However, the one enclosure that had stanchions in front to prevent visitors from interacting with the glass saw substantially fewer visitor-glass interactions during the Zoo Open period than the other enclosures observed. Although not a universal reaction, the change in European glass lizards’ behavior suggests that visitor factors such as density and behavior should be explored further, and mitigation should potentially be considered for a greater number of glass-fronted enclosures.

A fair number of the reptiles assessed in this study, such as the beaded lizards, had a neutral or intermediate response to visitors. Although their response may be due to their natural ecology and the traits they have developed to thrive, we also have to consider whether their lack of response was due to methodological limitations. We did not track environmental factors such as temperature in the building or temperature gradients in the enclosures, which may have affected behavior and space use. Riley et al. [26] found a relationship between the behavior of Nile crocodiles and temperature that could be a confound when looking at visitor effects. The extraneous light from skylights and windows also varied throughout the study and may have impacted seasonal behaviors, such as the increased social behaviors seen in the beaded lizards and Catalina Island rattlesnakes when the zoo was open. In addition, reptiles come with a unique set of observational challenges because some are inactive for substantial amounts of time (>90% of the time). This inactivity could be a natural component of their activity budget or potentially related to observational periods not synced with the reptiles’ active periods. More intensive data collection throughout a 24 h period is necessary to holistically assess changes in reptile welfare. Carter et al. [27] assessed visitor impact on tokay geckos and compared low and high sampling intensity. Their results showed a stronger impact of visitors with the higher sampling intensity. As such, the absence of detectable behavioral change does not necessarily translate to lack of impact on welfare. We also had a limited number of individuals for each species sampled, making generalizations of the data for the species observed impossible. This study was also performed with mature animals with years of previous visitor experience. Furthermore, observations occurred during a period of restricted visitor numbers due to COVID-19 safety protocols, which potentially mitigated the influence of visitor presence. Finally, we need to consider if lack of behavioral change can be part of a self-fulfilling prophecy. As we do not expect reptiles to be behaviorally emotive, we either do not look for the right behaviors or we believe a lack of change indicates that a variable is not meaningful to the reptiles, rather than pushing for more intensive investigations to better understand what is meaningful to them.

Finally, the variability between species in response to visitor effects speaks to the need for further research to develop reptile welfare models similar to those designed for mammalian species. Queiroz and Young [61] created a predictive model to assess visitor impacts across multiple land-dwelling mammal species. The model considered habitat (open—animals from grasslands, closed—animals from forests), activity cycle (nocturnal, diurnal), diet (herbivore, omnivore, carnivore), and stratum use (terrestrial, arboreal) in the assessment of 17 mammal species. The model predicted that terrestrial, diurnal herbivores from closed habitats experienced more significant visitor effects than arboreal, nocturnal carnivores from open habitats. In line with this model, the Catalina Island rattlesnakes, which are nocturnal carnivores from open habitats, generally had neutral or positive changes in behaviors associated with visitor presence. In contrast, the Arrau turtles’ natural ecology of being diurnal omnivores from closed habitats positions them in the model towards increased impact by visitor presence. This did not seem to be the case. The turtles did increase their inactivity and decrease their evenness of space use after reopening, but they also increased their behavioral diversity, providing evidence for a more neutral visitor impact. The European glass lizards seem to have been the most impacted by the presence of visitors with a decrease in SPI and time spent exposed when visible, but the model would suggest that as diurnal, terrestrial carnivores, they should have had an intermediate response between the Catalina Island rattlesnakes and Arrau turtles. These differences in the expected results could be due to factors that are important to non-mammalian taxa not being included initially in the Queiroz and Young model. Aquatic and aerial animals may be impacted by visitors differently than land-dwelling animals. In addition, fossorial or semi-fossorial animals may respond to visitor-related stimuli differently than other species in a broad “terrestrial” category. Other factors such as predator-defense mechanisms and the design of the captive habitat could also influence the impact of visitors and subsequently the welfare of animals in a captive environment.

## 5. Conclusions

COVID-19 temporary closures offered the zoo community the opportunity to broaden the field of visitor effects, but further research is still needed. The results in this study add to the growing evidence that the impacts of visitors vary across species, potentially due to predispositions of species based on their natural ecologies. They also highlight the benefits of more inclusive animal behavior models that consider factors important to non-mammalian species. A more comprehensive understanding of the behavioral responses of reptiles to visitors and factors that mitigate these responses can ensure that animals living at zoos have opportunities to experience optimal welfare.

## Figures and Tables

**Table 1:** Summary of natural ecology factors for each species.

Species	Habitat	Activity Cycle	Diet	Stratum Use
Catalina Island Rattlesnake (*Crotalus catalinensis)*	Open [4]	Nocturnal [7]	Carnivore [4]	Terrestrial [4]
European Glass Lizard (*Pseudopus apodus*)	Closed [8]	Diurnal [8]	Carnivore [8]	Terrestrial [8] (semi-fossorial)
Beaded Lizard (*Heloderma horridum*)	Closed [9]	Diurnal [9]	Carnivore [9]	Terrestrial [9] (arboreal)
Sonoran Spiny-tailed Iguana (*Ctenosaura macrolopha*)	Open [5]	Diurnal [30]	Omnivore [12]	Terrestrial [5] (arboreal)
Arrau Turtle (*Podocnemis expansa*)	Closed [13]	Diurnal [31]	Omnivore [13]	Aquatic [13]
Dwarf Caiman (*Paleosuchus palpebrosus*)	Closed [6]	Nocturnal [32]	Carnivore [11]	Aquatic [6]

**Table 2:** Summary of enclosure, species, and individual information.

Enclosure	Species	Inhabitants Sex	Ages at Start of Study	Area of Enclosure	Visual of the Enclosure
A	Catalina Island Rattlesnake	Male Female	6 years 4 years	2.3 m^2^	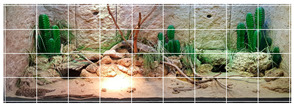
B	European Glass Lizard	Male Female Female Female	22 years 22 years 22 years 25 years	2.2 m^2^	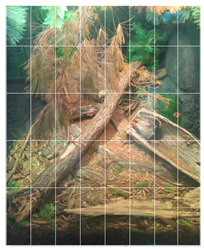
C	Beaded Lizard	Male Female Unknown	26 years 9 years 25 years	4.6 m^2^	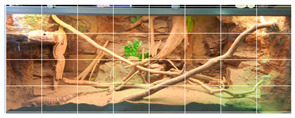
	Sonoran Spiny-tailed Iguana	Male	10 years		
D	Arrau Turtle	Male Female	39 years 39 years	17.3 m^2^	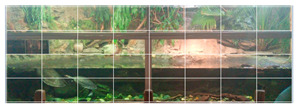
	Dwarf Caiman	Male Female	34 years 28 years		

**Table 3:** Summary of Behavioral Observations.

Time Period	Enclosures	Condition of the Zoo	Approximate Number of Hours Observed	Total Number of Days Analyzed	Comment
20 April 2020 to 14 May 2020	A, B, C	Closed	9 h	A—18 B—18 C—18	
12 May 2020 to 5 June 2020	D	Closed	8 h	D—16	An enclosure modification on 11 May 2020, caused a shift in the observational period.
15 June 2020 to 9 July 2020	All Enclosures	Open	9 h	A—18 B—17 C—18 D—18	

**Table 4:** Behavioral ethogram modified from Rose et al. [36] and Spain et al. [37]. All behaviors were included in scan sampling, and behaviors marked with an asterisk (*) were also recorded using all-occurrence sampling.

Behavior	Definition
Startle/Freeze *	Sudden cessation of movement accompanied by muscle tension while remaining frozen in place
Interact with Transparent Boundaries (ITB) *	Repetitive movement directed along glass boundaries of the enclosure; must complete the same circuit at least three times to count as ITB
Social *	Any interaction involving two or more animals
Move	Whole-body movement that causes a change in location, either via walking, undulating, swimming, or other means
Eat	Consumption of food or water
Investigate	Movement of the head and neck region to touch or nearly touch features in the immediate surroundings; includes tongue-flicking for snakes and lizards
Inactive	The individual is stationary
Other	Any behavior not previously described that involves whole-body movement
Not Visible	The individual or the individual’s behavior cannot be seen
**Exposure**	**Definition**
Hiding	More than half of the body is concealed from the observer under substrate or furnishings
Exposed	Less than half of the body is concealed from the observer under substrate or furnishings

**Table 5:** Wilcoxon Two-Sample with Monte Carlo Exact Test Results for behavior variables. Significant differences (Pr > |S-Mean| below 0.05) are bolded with an asterisk (*), and Pr > |S-Mean| equal to 0.01 or below are bolded with a double asterisk (**).

Species	Behavior	Percent of Time				Statistic (S)	Z-Score	Pr > |S-Mean|
		(Mean ± SE)		Median (Range)				
		Zoo Closed	Zoo Open	Zoo Closed	Zoo Open			
Catalina Island Rattlesnake	Visibility	99.61 ± 0.00	99.92 ± 0.08	100 (93.0–100)	100 (97.0–100)	1295.00	−0.62	0.49
	Social **	0.37 ± 0.25	2.41 ± 0.70	0.00 (0.00–3.33)	1.67 (0.00–10.00)	1050.00	−3.68	<0.01
	Investigate *	0.09 ± 0.09	0.93 ± 0.19	0.00 (0.00–3.33)	0.00 (0.00–10.00)	1187.50	−2.48	0.03
	Inactive **	99.17 ± 0.46	95.36 ± 1.12	100 (90.00–100)	96.70 (63.3–100)	1640.00	4.20	<0.01
	Exposed	38.70 ± 22.78	40.09 ± 11.94	10.00 (0.00–100)	50.00 (0–100)	1287.50	−0.30	0.77
European Glass Lizard	Visibility **	81.81 ± 3.49	57.91 ± 4.03	85.00 (62.5–100)	51.72 (28.33–90.83)	199.50	−3.52	<0.01
	Social	0.00 ± 0.00	0.00 ± 0.00	0.00 (0.00)	0.00 (0.00)	306.00	0.00	1.00
	Investigate	1.67 ± 0.40	2.88 ± 1.40	1.09 (0–0.06)	0.00 (0–23.53)	284.00	−0.75	0.46
	Inactive	92.73 ± 3.47	89.23 ± 1.24	94.67 (81.9–100)	94.29 (52.94–100)	309.00	0.10	0.93
	Exposed **	34.40 ± 3.57	13.09 ± 2.67	37.50 (9.17–57.50)	12.71 (0–42.98)	194.00	−3.70	<0.01
Beaded Lizard	Visibility	98.77 ± 1.23	100.00 ± 0.00	100 (50.00–100)	100 (100)	2889.00	−1.42	0.49
	Social *	0.50 ± 0.25	2.72 ± 1.09	0.00 (0.00–6.90)	0.00 (0.00–33.33)	2700.50	−2.35	0.02
	Investigate	3.47 ± 0.61	5.15 ± 3.33	0.00 (0.00–46.67)	0.00 (0.00–48.28)	2833.50	−0.80	0.43
	Inactive	93.74 ± 0.50	88.97 ± 4.20	100 (50.00–100)	100 (34.48–100)	3116.00	1.20	0.23
	Exposed	87.84 ± 6.12	98.39 ± 0.50	100 (0.00–100)	100 (83.33–100)	2945.50	0.02	0.99
Sonoran Spiny-tailed Iguana	Visibility	100.00 ± 0.00	100.00 ± 0.00	100 (100)	100 (100)	333.00	0.00	1.00
	Social	0.00 ± 0.00	0.00 ± 0.00	0.00 (0.00)	0.00 (0.00)	333.00	0.00	1.00
	Investigate	0.19 ± 0.19	0.94 ± 0.46	0.00 (0.00–3.44)	0.00 (0.00–6.90)	307.00	−1.37	0.28
	Inactive	97.38 ± 1.15	96.62 ± 1.31	100 (82.76–100)	100 (82.76–100)	338.00	0.19	0.85
	Exposed **	99.81 ± 0.19	95.35 ± 2.72	100 (96.67–100)	98.33 (50.00–100)	407.00	2.98	<0.01
Arrau Turtle	Visibility	99.69 ± 0.10	99.72 ± 0.09	100 (96.67–100)	100 (96.67–100)	1098.00	−0.15	1.00
	Social	1.05 ± 0.003	1.95 ± 0.09	0.00 (0.00–3.45)	0.00 (0.00–6.67)	993.50	−1.57	0.12
	Investigate	7.21 ± 3.04	9.91 ± 3.42	3.33 (0.00–30.00)	6.67 (0.00–43.33)	1056.50	−0.59	0.56
	Inactive **	5.97 ± 3.70	39.89 ± 1.60	3.33 (0.00–20.00	40.00 (0.00–100)	660.00	−5.48	<0.01
	Exposed	99.79 ± 0.00	99.54 ± 0.09	100 (96.67–100)	100 (96.67–100)	1148.00	1.03	0.43
Dwarf Caiman	Visibility	99.79 ± 0.00	99.81 ± 0.19	100 (96.67–100)	100 (96.67–100)	1085.00	−0.09	1.00
	Social	0.00 ± 0.00	0.09 ± 0.09	0.00 (0.00)	0.00 (0.00–3.33)	1072.00	−0.96	1.00
	Investigate	0.21 ± 0.004	0.10 ± 0.10	0.00 (0.00–3.45)	0.00 (0.00–3.33)	1107.50	0.68	0.48
	Inactive **	98.12 ± 0.83	99.81 ± 0.01	100 (86.67–100)	100 (96.67–100)	940.00	−2.78	<0.01
	Exposed	99.27 ± 0.31	96.59 ± 2.67	100 (83.33–100)	100 (0.00–100)	1116.00	0.62	0.60

**Table 6:** Spearman correlation coefficients for the relationships between visitor-glass interactions and visibility and time exposed when visible. Significant differences of *p* ≤ 0.01 are bolded with a double asterisk (**). Values marked N/A indicate there was not sufficient variation in the measure to test a given correlation.

Enclosure	Daily Observation Rate of Visitor-Glass Interaction during Zoo Open		Species	Visibility	Exposed
	Mean ± SE	Median & Range			
A	7.51 ± 1.15	7.50 (0.00–16.00)	Catalina Island Rattlesnake	r = 0.07 *p* = 0.57 *n* = 72	r = −0.03 *p* = 0.83 *n* = 72
B	6.02 ± 0.91	6.00 (0.00–12.10)	European Glass Lizard	r = 0.46 *p* = <0.01 ** n = 35	r = −0.55 *p* = <0.01 ** n = 35
C	7.56 ± 1.08	6.50 (0.00–17.00)	Beaded Lizard	r = 0.12 *p* = 0.23 *n* = 108	N/A
			Sonoran Spiny-tailed Iguana	N/A	N/A
D	0.06 ± 0.06	0.00 (0.00–1.00)	Arrau Turtle	N/A	N/A
			Dwarf Caiman	N/A	N/A

**Table 7:** Wilcoxon Two-Sample with Monte Carlo Exact Test Results for Shannon–Wiener Index (H). Significant differences of Pr > |S-Mean| equal to 0.01 or below are bolded with a double asterisk (**).

Species	Shannon-Wiener Index				Statistic (S)	Z-Score	Pr > |S-Mean|
	**Mean ± SE**		**Median (Range)**				
	**Zoo Closed**	**Zoo Open**	**Zoo Closed**	**Zoo Open**			
Catalina Island Rattlesnake **	0.04 ± 0.02	0.16 ± 0.03	0.00 (0.00–0.13)	0.13 (0.08–0.29)	43.00	−2.67	**<0.01**
European Glass Lizard	0.23 ± 0.02	0.25 ± 0.05	0.22 (0.21–0.28)	0.27 (0.13–0.34)	18.00	0.00	1.00
Beaded Lizard	0.21 ± 0.01	0.30 ± 0.06	0.19 (0.00–0.37)	0.31 (0.00–0.59)	134.00	−0.83	0.37
Sonoran Spiny-tailed Iguana	0.11 ± 0.04	0.13 ± 0.03	0.09 (0.03–0.23)	0.11 (0.07–0.22)	16.00	−0.58	0.69
Arrau Turtle **	0.41 ± 0.06	0.69 ± 0.01	0.44 (0.12–0.58)	0.67 (0.50–0.96)	39.00	−3.05	**<0.01**
Dwarf Caiman **	0.07 ± 0.03	0.01 ± 0.01	0.07 (0.00–0.15)	0.00 (0.00–0.04)	90.00	2.47	**0.01**

**Table 8:** Wilcoxon Two-Sample with Monte Carlo Exact Test Results for Dickens’ Spread of Participation Index (SPI). Significant differences (Pr > |S-Mean| below 0.05) are bolded with an asterisk (*), and Pr > |S-Mean| equal to 0.01 or below are bolded with a double asterisk (**).

Species	Dickens’ Spread of Participation Index				Statistic (S)	Z-Score	Pr > |S-Mean|
	Mean ± SE		Median (Range)				
	Zoo Closed	Zoo Open	Zoo Closed	Zoo Open			
Catalina Island Rattlesnake	0.91 ± 0.02	0.83 ± 0.01	0.92 (0.82–1.00)	0.83 (0.69–0.92)	83.00	1.58	0.12
European Glass Lizard *	0.55 ± 0.03	0.80 ± 0.06	0.56 (0.48–0.60)	0.77 (0.69–0.98)	10.00	−2.31	**0.03**
Beaded Lizard	0.88 ± 0.01	0.91 ± 0.01	0.88 (0.79–0.94)	0.93 (0.78–0.97)	121.00	−1.68	0.10
Sonoran Spiny-tailed Iguana	0.86 ± 0.02	0.80 ± 0.03	0.87 (0.79–0.89)	0.80 (0.73–0.86)	23.00	1.44	0.20
Arrau Turtle **	0.58 ± 0.005	0.63 ± 0.02	0.58 (0.57–0.60)	0.63 (0.60–0.69)	39.00	−3.05	**<0.01**
Dwarf Caiman	0.81 ± 0.03	0.88 ± 0.02	0.78 (0.67–0.97)	0.88 (0.83–0.94)	53.00	−1.58	0.12

## Data Availability

Data is available upon reasonable request.
